# Measuring nanoparticles in the size range to 2000 nm

**DOI:** 10.1007/s11051-018-4397-x

**Published:** 2018-12-06

**Authors:** Philip J. Wyatt

**Affiliations:** Wyatt Technology Corporation, 6330 Hollister Avenue, Goleta, CA 93117 USA

**Keywords:** Rods, Spheres, Gold, Aggregates, Carbon nanotubes, Particle suspensions

## Abstract

Measurement of light scattered from suspensions of monodisperse nanoparticles in solution (“turbidity”) long has been used to derive their size. Following some means of fractionation, the light (monochromatic) scattered by the particles into a set of distinct angles is collected and a non-linear least squares fit was made to an appropriate theory in order to extract their size. For a wide range of particle structures, where this process becomes very complex and of questionable validity, there is a far simpler interpretive means based upon measurements at extremely small, and often inaccessible, scattering angles. A method is described whereby the required small angle values are derived from measurements made over a range of larger, more readily accessible, angles. Although the basis for the analyses developed is the Rayleigh-Gans approximation, the results presented confirm that the method provides meaningful results up to a size of about 2000 nm. The larger sizes are well beyond the RG limits.

## Introduction

The measurement of light scattered from a suspension of particles (often referred to as a turbidity measurement) has been used historically (Koch 1961) as a means to determine their average size. In a practical sense, the incident light is monochromatic and well collimated, such as it might be produced by a laser. For specific size determinations, the particles must be monodisperse (in size and structure) and the scattered light intensities were collected over a range of angles with respect to the direction of the incident illumination. However, even for a monodisperse solution of particles, the determination of their size and a measure of their structural features require additional information. A particle “model” is generally required, i.e., what are the known structural properties of the particles being measured? Are the particle homogeneous spheres? Are they rods? Ellipsoids? Radially symmetric spheres? Other structures? Conceptually, it would be ideal if the particle size and structure could be determined from the collected scattered intensities alone. (Traditionally, scattered light intensities are collected at an explicit set of angles and, relative to the direction and polarization of the incident monochromatic beam, polarizations. Associated with each intensity value collected is its experimental standard deviation.) Deriving the particle structural properties associated with these measurements is generally referred to as solving the “inverse scattering problem,” a subject of continuing study.

## The Lorenz-Mie theory and the Rayleigh-Gans approximation

From a practical point of view, the structure of the scattering particles is known a priori so that the collected data might be used to extract the “best fit” structural parameters. For example, if the particles are known to be homogeneous spheres in a medium of refractive index *n*_0_, one could use such a best fit to the Lorenz-Mie (LM) scattering theory (Lorenz 1890, 1898; Logan 1965; Mie 1908) to extract a best-estimate of the radius *a* and the refractive index of the sphere, *n*. This exact theoretical model has been extended to a broad variety of spherically symmetric structures (Aden and Kerker 1951; Kerker 1988; Wyatt 1962). For more complex structures such as ellipsoids of revolution, a far more complex scattering theory must be employed that includes the orientation of the ellipsoid’s axes with respect to the incident beam direction. From an ensemble of identical ellipsoids, a best fit would have to be extracted from a model that includes averaging over all possible orientations. Even a simple homogeneous sphere of refractive index *n*_1_ and radius *a* with a homogeneous coating of refractive index *n*_2_ and thickness *t* will require a complex set of computations (Aden and Kerker 1951) to extract the parameters *n*_1_, *n*_2_, *a*, and *t* that correspond to a best fit to the data.

The scientific literature is filled with myriad articles (Kerker 1988) describing elements of “Forward Scattering” theory, i.e., given its structure, how does a particle scatter light? Sometimes, models based on exact theory are used, while others may involve iterative approximations to such exact theory. The so-called Rayleigh-Gans (Debye) *approximation* (Kerker 1969; Newton 1982; Van de Hulst 1981) appears to be of greatest practical importance as it permits a relatively simple interpretive formalism. This approximation is the optical equivalent of the Born approximation, familiar in classical quantum mechanics (Schiff 1955) scattering theory. The potential *V* of quantum mechanics is replaced by a constant relative refractive index, *m* = *n*/*n*_0_, throughout the region occupied by the scattering particle where the particle is of refractive index *n* in a medium of refractive index *n*_0_. In the Born approximation, the scattering of neutrons and x-rays from a variety of nuclei is described by the same expressions (Feigan et al. 1987). Such neutron and x-ray scattering measurements often rely on low-angle scattering data to derive scattering structures. The particles that scatter the incident neutrons and x-rays are usually assumed to be highly tenuous; very much as the scattering particles in the RG approximation are assumed barely differentiable in a medium of closely matched refractive index.

Particles for which RG is most frequently applied to extract their size are characteristically suspended in water or other fluids whose polarizability is comparable to that of the particles themselves. Thus *n* ≈ *n*_0_ and, therefore, *m* ≈ 1. In that case, we require (*m*^2^ − 1)/(*m*^2^ + 2) ≃ (*m* + 1)(*m* − 1)/(*m*^2^ + 2) ≃ 2(*m* − 1)/3 ≃  ∣ *m* − 1 ∣ ≪ 1. In effect, this condition implies that the polarizability of the particle and its surrounding medium are nearly the same. In addition, the RG approximation requires that the phase shift, *ρ*, of the incident light as it passes through the particle be very small, i.e*.*, *ρ* = 2*ka* ∣ *m* − 1 ∣ ≪ 1, where *k* = 2*π*/*λ* = 2*πn*_0_/*λ*_0._ From such light scattering measurements, the size and, sometimes, other structural details of the particles are, hopefully, derived. This exercise is a very simple example of the “inverse scattering problem” (Colton and Kress 1998; Cakoni and Colton 2010) whereby from the collected scattering data, scattering particles may be characterized or even identified. For such analyses, the shape and structure of the particles are assumed to be known and only their associated dimensions deduced. As discussed above and in further detail in an earlier paper (Wyatt 1993), the general “requirements” for application of the RG approximation are stated frequently as the following:

1 $$ \mid m-1\mid \ll 1 $$

2 $$ 2 ka\mid m-1\mid \ll 1. $$

If ∣*m* − 1∣ is not ≪ 1, measurements at very small scattering angles *θ* still *might* be useful if

3 $$ 2 qa\mid m-1\mid \ll 1, $$

where *q* = 2*k* sin(*θ*/2). Thus, by restricting measurement of scattered light to very small scattering angles, the RG approximation *might* provide a means to derive a particle size per Eq. (3). This will be discussed further in the “Analytical extension: the form factor at very small angles” section.

Modern light scattering instrumentation is designed to collect scattered light at detectors placed at discrete angles within the range 0 < *θ* < 180^∘^. A typical experiment consists of measurement of light scattered over that range of scattering angles (referred to as *differential light scattering* or now, more commonly, as multiangle light scattering or MALS) and, depending upon the sample measured, may not even include sufficiently small scattering angles for which Eq. (3) might be valid. Similar measurements over a range of scattering angles are common, as well, for the interpretation (Feĭgin et al. 1987) of x-ray and neutron (Wyatt et al. 1960) scattering experiments, though scattering measurements are frequently restricted to small angles.

One of the major areas for the successful application of the RG approximation has been in the fields of polymer and protein chemistry. Here, the “particles” are polymer and protein molecules whose sizes are characteristically very small compared to the wavelength of the incident radiation. It is important to note, however, that the main focus of polymer and protein measurements based on light scattering (Huglin 1972; Zimm 1948) is the determination of molar masses and interactive properties of the molecules. When the particle/molecule size is smaller than about 20 nm, which is most often the case, the angular variation of the scattered intensities may be too small to derive a size.

As discussed by Zimm (1948a, 1948b) and others (Huglin 1972; Wyatt 1993), the variation of scattered light [the Rayleigh ratio, *R*(*θ*)] from a sample of volume V at very low concentration is proportional to a corresponding form factor, *P*(*θ*), characteristic of the particles present in the illuminated sample. *P*(*θ*) is also referred to as the particle scattering function. Thus for incident vertically polarized light of intensity *I*_0_, the scattered intensity, *I*(*θ*), is given by

4 $$ R\left(\theta \right)=\frac{I\left(\theta \right)}{I_0}=\frac{(ka)^4{V}^2}{4\pi }{\left|m-1\right|}^2P\left(\theta \right). $$

As an example, consider an ensemble of identical cylindrical rods of length *L* and radius *a*. We must average over all their orientations,*α*, with respect to the direction of the incident light to yield the RG approximation (Kerker 1969; Elicabe 2010)

5 $$ P\left(\theta \right)=\underset{0}{\overset{\frac{\pi }{2}}{\int }}{\left[\frac{2{J}_1\left( qa\sin \alpha \right)}{qa\sin \alpha}\frac{\sin \left( qL\cos \alpha /2\right)}{qL\cos \alpha /2}\right]}^2\sin \alpha d\alpha . $$

In the limit of an extremely thin rod (*a* → 0), we have the further approximation

6 $$ P\left(\theta \right)=\frac{1}{x}\underset{0}{\overset{2x}{\int }}\frac{\sin v}{v} dv-{\left(\frac{\sin x}{x}\right)}^2, $$

where *x* = (2*πL*/*λ*) sin(*θ*/2) = *kL* sin(*θ*/2).

A more frequently seen example of the RG approximation is that for homogeneous spheres of radius *a* for which

7 $$ P\left(\theta \right)={\left[\frac{3}{u}{j}_1(u)\right]}^2, $$

where *u* = 2*ka* sin(*θ*/2) = *qa* and *j*_1_(*u*) is the spherical Bessel function of order unity. It may be shown that *P*(0) = 1 and 0 ≤ *P*(*θ*) ≤ 1.

Scattering data, collected at a set of discrete angles, are then used to make a non-linear least squares fit to Eq. (5), (6), or (7) to extract the best value of the length *L* for the rods or the radius *a* for the spheres. For Eq. (5), the value of the cylinder radius *a* is assumed to be known from prior microscopy measurements. Similar expressions have been developed for a variety of other axially symmetric structures including tubes, ellipsoids, superellipsoids, rings, and disks. Such analyses are by no means easy, but compared to similar fitting analyses to the full Lorenz-Mie theory, they are much simpler. It is emphasized, however, that the fits of the RG approximations, such as the three listed above, are assumed to be meaningful only if the particles are suitably described by Eqs. (1) and (2). Non-linear least squares analyses based explicitly on Eq. (5) are rarely, if ever, seen.

## Limitations of RG

In 1963, Kerker et al. (1963) published a rarely referenced paper in which they compared the RG approximation for a sphere, Eq. (7), to the exact Lorenz-Mie solution. Although their main purpose was to study the limits of the RG approximation for a sphere, they actually addressed a much more general question: How reasonable is *any* use of the RG approximation to obtain meaningful interpretations of scattering data from structures characterized by limits expressed in Eqs. (1) and (2)? Obviously, if the approximation had limitations for so well-described particles as homogeneous spheres, applying it to other, perhaps more complex, structures could not be expected to provide any significant expectations of obtaining more meaningful results. For their calculations, the authors consider a broad range of sizes (0 ≤ *x* ≤ 12) relative to the incident wavelength, i.e., *x* = *ka* = 2*πa*/*λ* where *λ* = *λ*_0_/*n*_0_ as well as a broad range of relative refractive indices, *m,*1 ≤ *m* ≤ 2. They compared the RG results directly for the total scattering cross sections as well as specific scattered intensities at a few selected angles (10^∘^, 20^∘^, and 45^∘^). The most significant conclusions of their study were summarized in their comments “…The (RG) approximation is best for small values of *m* − 1 and small angles (forward direction)…. The agreement between RG… and (Lorenz-) Mie is very poor except for the smallest values of x….”

The RG approximation is commonly applied only for small particles of simple structure and, even then, the results reported may be wrong. It may well have been overlooked by Kerker et al. (1963); however, their calculations suggest that in regions where Eq. (3) applies, meaningful sizes might be extracted if scattering results at extremely small scattering angles *θ* could be derived. Therein lays the problem, of course, as measurements at small scattering angles are prone to scattering from sample and solution contaminants which tend to overwhelm the scattering by the sample particles themselves, especially if the particles of interest are of radii much smaller than the wavelength of the incident light.

**Fig. 1:**
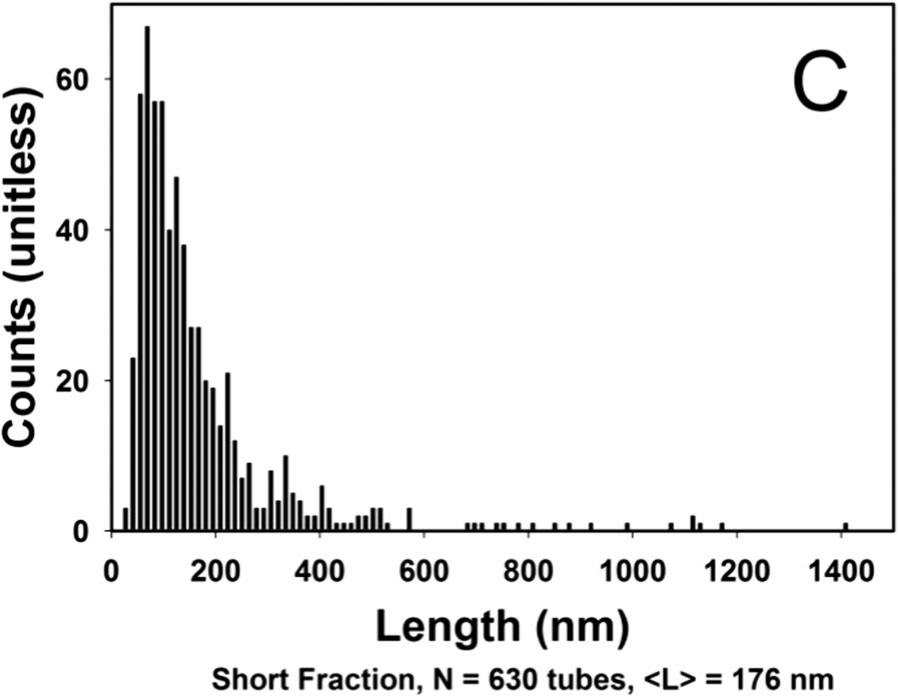
Size distribution of 630 measured short fraction rods of NIST reference set

Consider now an example where both Eqs. (1) and (2) would appear to exclude any possibility of RG providing a measure of particle size since the refractive index is far beyond any reasonable value: a gold particle of radius 250 nm and refractive index 0.14246 + 3.6821*i*. Figure 1 contrasts the exact Lorenz-Mie theory at the wavelength of 658 nm with the RG approximation of Eq. (7) for the case of such spheres in water *n*_0_ = 1.33. For gold at that wavelength, the value of ∣*m* − 1∣ = 4.99. The data at the 15 angles indicated by the superimposed cross hatches correspond to the set that would be measured with a commercial MALS instrument (DAWN HELEOS II n.d.) as listed explicitly in Table 1, below.

**Table 1:** Scattering angles measured for aqueous medium *λ* = 664 nm

Detector	Angle	sin^2^(θ/2)
1	13°	1.28 × 10^−2^
2	20.7°	3.23 × 10^−2^
3	29.6°	6.33 × 10^−2^
4	37.5°	1.03 × 10^−1^
5	44.8°	1.45 × 10^−1^
6	53.1°	2.00 × 10^−1^
7	61.1°	2.58 × 10^−1^
8	70.1°	3.30 × 10^−1^
9	80.1°	4.14 × 10^−1^
10	90.0°	5.00 × 10^−1^
11	99.9°	5.86 × 10^−1^
12	109.9°	6.70 × 10^−1^
13	120.1°	7.51 × 10^−1^
14	130.4°	8.24 × 10^−1^
15	140.0°	8.83 × 10^−1^
16	149.0°	9.29 × 10^−1^
17	157.7°	9.63 × 10^−1^

Returning to the studies by Kerker et al. (1963) testing the applicability of the RG approximation to provide an accurate measure of the scattering by homogeneous spheres, we note that the authors inferred also a reasonable basis for estimating the applicability of the approximation to other particle shapes and forms. As we shall see later in this paper, the analytical extension of the RG approximation to very small scattering angles may permit a simplified means to extract a *reasonable* approximation of the size of such gold particles. If that is successful, we would expect that a similar approximation theory might well be used to determine structural properties of more complicated particles (with more common refractive indices!) for which an exact theory (such as Lorenz-Mie) does not exist. We shall return to these gold particle exemplars in the final section and confirm that the analytical procedure concept to be presented is both applicable and appropriate.

## Preparing and measuring selected samples

Before MALS measurements may be used to extract particle sizes and related features, appropriate *monodisperse* samples must be prepared. Samples of the selected particles are prepared in solution and then fractionated by size. For molecular species, or even small particles, fractionation may be achieved using columns based on size exclusion chromatography. For large particles, as well as smaller molecules, the preferred separation technique of asymmetric flow field flow fractionation (A4F) is used to obtain subsamples comprised of essentially identical particles. In the late 1980s, Carl-Gustav Wahlund (Wahlund and Giddings 1987; Wahlund and Litzén 1989) introduced the asymmetric flow concept as a simplification of the difficult to use, yet revolutionary, cross flow technique developed by J. Calvin Giddings (1966). Within a few years, Christoph Johann (2004) had perfected the system most frequently used based on a single pump. See also Jores et al. (2004). A rich literature covering the implementation of various types of FFF devices may be found in the FFF Bibliography (1965–2018). This listing of every known paper relating to field flow fractionation was developed and managed for many years by Dr. Mark Schure beginning when he worked for Rohm & Haas. The text by Podzimek (2011) should be consulted for further details of the A4F devices as well as size exclusion separation techniques and their applications. Using A4F, it is now possible to fractionate particles of unusual shapes and structures with relative ease within the very large range of sizes from 5 nm to several micrometers. Use of the A4F device has become the method of choice to fractionate such particles though, as we shall discuss in the section on “Examples of the analytical representation by *Π*_*m*_(*θ*)”, the mechanism by which rod-like particles are actually separated remains still an area of uncertainty.

Traditionally, with an a priori knowledge of the particle structures, a non-linear least squares fit of the MALS collected data is made to the LM theory for homogeneous spheres or, in the RG approximation, to Eq. (6), for thin rod-like structures, to extract the corresponding structural parameters such as the sphere radius *a* or thin rod length *L*. The latter assumes, of course, that scattering by such particles may be characterized by the RG approximation, i.e., satisfy Eqs. (1) and (2). For an ensemble of identical extremely thin rods in the RG approximation, a non-linear least squares fit of the measured MALS data to Eq. (6) would be expected to yield values of the rod length for each size fraction measured. The measured scattered intensity at each angle collected for this non-linear least squares fitting calculation is usually weighted by its reciprocal measured standard deviation. Thus, noisy data, associated with large standard deviations, are weighted less than more precisely measured data in performing the least squares analyses. The precision of the experimental measurements is critical to determining structural features of the scattering ensemble. Although these are complex calculations, the speed of even the most modest computer insures that the calculations are easily performed. Compared to fitting to the exact electromagnetic formalism, if such were available, the RG approximation is a much simpler analysis, though it is often only a poor representation of the scattering particles. Nevertheless, the process of setting up the formalism for each such type of structure may be time-consuming and difficult unless such calculations are routine; in general, they are not. Again, of course, for particles that do not satisfy Eqs. (1) and (2), there may be no suitable RG approximation for which the collected data may be used to estimate size.

**Fig. 2:**
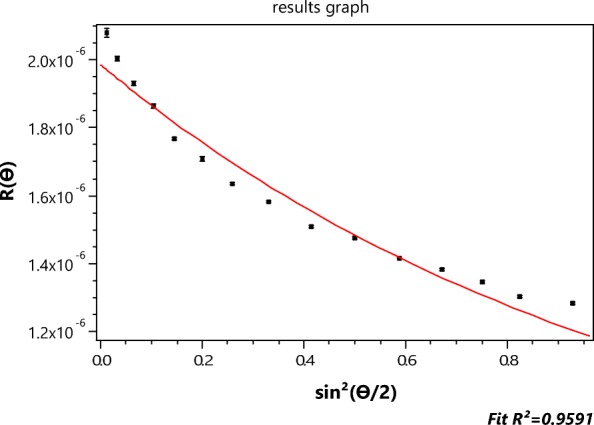
Best fit of the collected 15 angle scattering data to the RG rod model of Eq. (6); *R*(*θ*)*vs*. sin^2^(*θ*/2)

Following A4F fractionation of the sample in appropriately buffered aqueous solution (refractive index of 1.33), MALS measurements are made (Podzimek 2011) of each eluting fraction (often referred to as a “slice”) at the set of *n* discrete angles *θ*_*i*_, *i* = 1, …, *n* listed in Table 1. Often, because of poor calibration or for samples with large outlier particles, some angles are deleted during the subsequent analyses. A class of very important particles, where RG theory is applied frequently, is that of very thin rods such as cellulose (refractive index 1.47). Application of the RG result, Eq. (6) produces excellent fits to the measured data for some *cellulose* samples (Wyatt 2014). Whether or not such sizes are correct requires some form of ancillary measurement process such as examination by electron microscopic means; not easily done with such tenuous particles! On the other hand, when the approximation of Eq. (6) is applied to single-wall carbon nanotubes (SWCNT), it produces a poor fit and probably a poor estimate of their size. Although they are extremely thin rods of diameter about 1 nm, even smaller than cellulose tubes, application of Eq. (6) yields a very poor fit to the measured data, as we shall see presently in Fig. 2. Even though the refractive index of graphene (Wang and Nolte 2009) is very large *n* = 3 + 1.4*i*, the application of Eq. (6) to interpret the MALS measurements of these particles yields lengths that appear to be much shorter than those measured by electron microscopy. It should be pointed out, however, that the effective refractive index of SWCNTs is different from graphene. Indeed, considering such structures as water-filled tubes of wall thickness *t* and radius *a*, Wyatt (2014) shows an effective value of *n* per unit length as *n* = 2.08 + 0.625*i*. Thus in water *n*_0_ = 1.33 and *m* = 1.56 + 0.47*i*; therefore ∣*m* − 1 ∣  = 0.73. The wall thickness was assumed to be the diameter of a carbon atom (0.154 nm) and the tube diameter is generally taken as 1.2 nm. On this basis, a proper interpretation of the scattering by such structures should be based on application of Eq. (5); not an easy calculation.

In 2013, the National Institute for Standards and Technology (NIST) produced a Standard Reference Material 8281 comprised of dispersed single-wall carbon nanotubes of three length-resolved populations, each with broad distributions referred to as “short, medium, and long.” A detailed report of the associated investigation is discussed by Lin and Watters (2013). The size distributions of each of these fractions were obtained by measuring them manually one-at-a-time using transmission electron microscopy. Fractionation by centrifugation (Fagan et al. 2008) has also been used, but both techniques were difficult and relatively ineffective. Figure 1 shows the NIST tube length histogram as measured for 630 “short fraction” tubes.

**Fig. 3:**
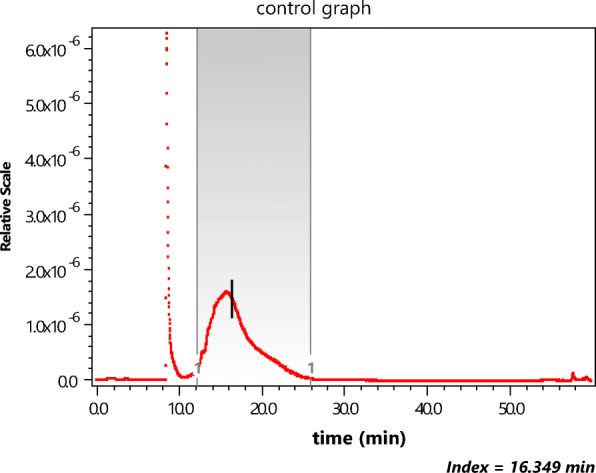
The scattered intensity at 90° from a suspension of the NIST short fraction as a function of elution time (seconds) during an A4F fractionation

In order to restrict the measurements to the shorter lengths, before injection into the A4F system, the NIST short samples were filtered through a 450-nm cellulose filter. An aliquot was then injected for fractionation by A4F, producing the elution of Fig. 3. The vertical bar shows the location of slice 979 whose MALS scattering data points are shown in Fig. 2.

**Fig. 4:**
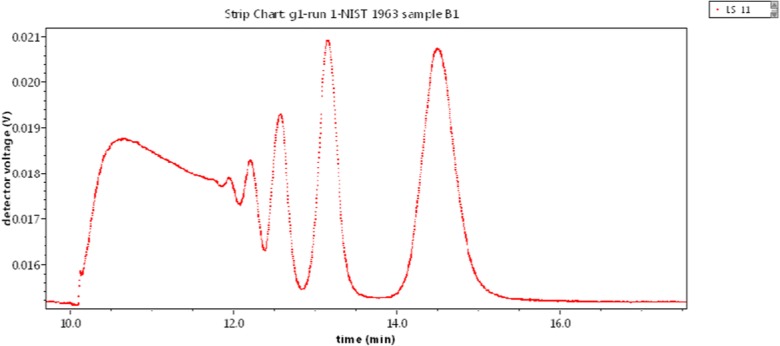
Latex sphere aggregates separated by disk centrifugation [intensity vs. time]

Figure 2 contrasts the (*n* = 15 detectors) data collected at slice 979 with their *best* fit to the RG rod model of Eq. (6) yielding a length of 189.4 ± 1.6 nm. The inapplicability of the RG approximation to such particles has been discussed earlier. Nevertheless, the literature is replete with the application of this model (cf Gigault et al. 2011) and the subsequent publication of the associated, possibly erroneous, results. The importance for measurement of these structures cannot be overemphasized. They, and similar objects, are the focus of much ongoing research in the field of *nanoparticles*, yet there are no known theoretical models to predict their light scattering and provide, thereby, a more precise basis for their measurement. Accordingly, one of the most important questions that this paper hopes to answer is: By what means might MALS measurements be used to extract particle shape and structural properties when an exact scattering theory for the particles of interest does not exist and application of the RG approximation may be inappropriate?

As to be presented later, there is a means to test the validity of Eq. (3) by which a reasonable size may be derived despite the failure of Eqs. (1) and (2). This analytical extension of the RG approximation to *derive* scattering data at very small angles is explained in further detail in the “Analytical extension: the form factor at very small angles” section.

Let us now examine a different class of structures comprised of various aggregates of 100-nm PSL spheres. Figure 4 presents the result of separation by means of a CPS disk centrifuge of the aggregated NBS1963 100-nm diameter polystyrene latex (PSL) standard (now referred to as NIST1963). Note that the most massive particles (aggregates) sediment most rapidly, i.e., the largest aggregates elute *first*, i.e., move most rapidly outward. Note that the rightmost peak must correspond to the unaggregated 100-nm diameter particles; the smallest particles present. What was once a standard suspension of single 100-nm PSL spheres had become a mixed suspension of various aggregates. Centrifugation separates by mass with the smallest fraction requiring the longest time to sediment and, for this aggregated sample, corresponding to the single, non-aggregated spheres. Next would be doublets, then triplets, etc. Although some work had begun whereby MALS measurements were combined with centrifugal separation (Wyatt 2010), the instrumentation at the time was limited to the three scattering angles 20°, 60°, and 80°. It remains a “work in progress.”

**Fig. 5:**
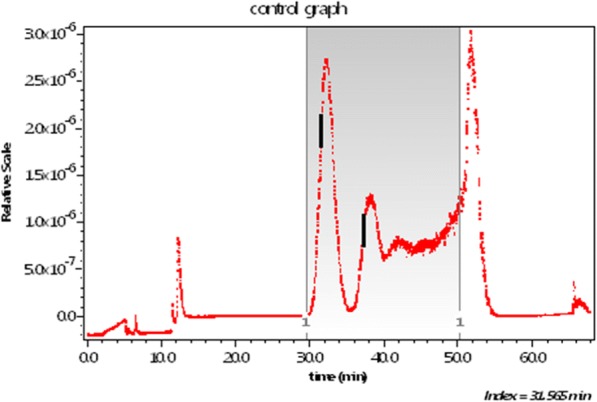
Latex sphere aggregates separated by A4F [*I*(*θ*) vs. time]

**Fig. 6:**
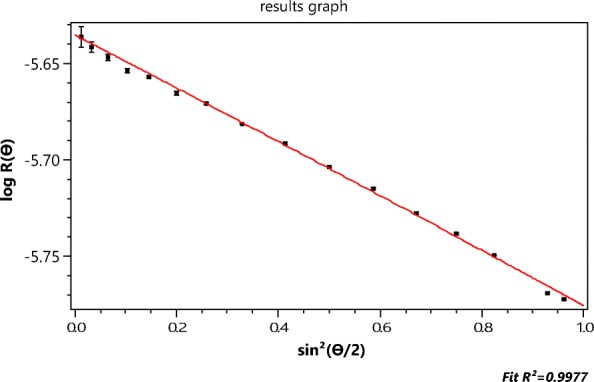
Fit of the slice 1876 data to the LM theory [log*R*(*θ*) vs. sin^2^*θ*/2]

An aliquot of the aggregated NBS 1963 sample shown in Fig. 4 was then separated by A4F producing the fractogram shown in Fig. 5, below. Note the two peaks clearly resolved in that figure. They should correspond to the smallest particle groups: Monomer and dimer of the original sample. The vertical bar at an elution time of about 32 min corresponds to Slice 1876 in the monomer of this elution. Slice 2193, at an elution time of about 37 min, lies in the dimer peak region, is also marked. The collected data of slice 1876 and their 16-angle fit to the LM theory are shown in Fig. 6. The LM theory yields a radius of 48.9 ± 0.3 nm for this slice while the RG sphere model [Eq. (7)] yields 49.9 ± 0.4 nm. The refractive index of PSL at the wavelength of 664 nm is 1.59 while that of water is approximately 1.33. For these small latex spheres, this RG approximation is excellent. As mentioned, the second peak of Fig. 5 corresponds to the dimer, so we might try to establish an equation like Eq. (5) for a double touching sphere structure averaged over all orientations for the RG approximation and deduce its component sphere sizes. Such an analysis and calculation would be difficult. An earlier paper (Wyatt 2014) presents some details relating such multiple sphere aggregates to the size of each contributing identical sphere. An exact representation of the scattering by two spheres has been developed by Fuller et al. (1986), but the calculations required to extract the sphere sizes are also complex. There is a far simpler means, however, derived from the RG approximation as will be shown later.

## The relation between the mean square radius and the form factor, *P*(*θ*)

As mentioned earlier, most commercial light scattering instrumentation is designed to collect scattered light at detectors spanning a broad range of angles. Yet application of Eq. (3), for example, requires measurements at very small angles. Even if very small angle measurements were available, the means for extracting size information therefrom is not clear. The major objective of this paper is to show how traditional large angle measurements may be used as a basis for deriving key nanoparticle dimensions especially in regions where the RG approximation of Eqs. (1) and (2) may fail.A result of particular importance for light scattering, and especially related applications of the RG approximation, arose from studies of polymer molecules. During the developments by Zimm (1948a, 1948b) and others (Huglin 1972) of the relation of scattered light measurements and molar mass, an associated “size” was obtained from these same measurements: the molecule’s mean square radius, $\left\langle {r}_g^2\right\rangle .$ As presented very clearly by Kratochvíl (1987), a homogeneous molecule/particle of mass *M* is assumed to be comprised of *n* identical elements of mass *m*_*i*_. Its associated mean square radius is defined as

8 $$ \left\langle {r}_g^2\right\rangle =\frac{1}{2{n}^2}\sum \limits_{i=1}^n\sum \limits_{j=1}^n\left\langle {h}_{ij}^2\right\rangle, $$

where *h*_*ij*_ is the distance between the *i*^th^ and *j*^th^ mass element and $\left\langle {h}_{ij}^2\right\rangle$ is the square of this distance averaged over all conformations. Eq. (8) may be shown to reduce to the more familiar form

9 $$ \left\langle {r_g}^2\right\rangle =\sum \limits_i{m}_i{r}_i^2/\sum \limits_i{m}_i=\sum \limits_i{m}_i{r}_i^2/M, $$

where *M* is the particle’s total mass and *r*_*i*_ is the distance of the *i*th element from the particle’s *center of mass* (the subscript “*g*” referring to the center of *gravity*, i.e., center of mass). This same structural parameter is derived similarly for the interpretation of small angle x-ray and neutron scattering (Feĭgin et al. 1987) from various particles some of whose structural features are to be derived from such measurements. Note that Eqs. (8) and (9) are *not* approximations nor do they depend on any theoretical limitations. Thus irrespective of shape, refractive index, or size, the mean square radius, $\left\langle {r}_g^2\right\rangle,$ for most particle structures may be calculated from data collected over a range of scattering angles as will be shown below.

Returning to Eq. (9) for a homogeneous molecule/particle of volume *V*, density *ρ*, and mass *M* = *ρV*, we obtain Eq. (10) where *m*_*i*_ = *ρv*_*i*_ and *R*(*r*, *θ*, *φ*) is the distance of the mass

10 $$ \left\langle {r}_g^2\right\rangle =\frac{\sum {m}_i{r}_i^2}{M}=\frac{\sum {v}_i{r}_i^2}{V}=\frac{1}{V}\iiint {R}^2\left(r,\theta, \varphi \right) dv, $$

element *ρdv* from the particle center of mass. For a homogeneous sphere of radius *a*, for example, Eq. (10) yields

11 $$ \left\langle {r}_g^2\right\rangle =\frac{1}{V}\iiint {r}^2 dV=\frac{3}{4\pi {a}^3}\underset{0}{\overset{2\pi }{\int }} d\varphi \underset{0}{\overset{\pi }{\int }}\sin \theta d\theta \underset{0}{\overset{a}{\int }}{r}^4 dr=\frac{3}{5}{a}^2. $$

For this simple example, the center of mass lies at the center of the sphere.

The mean square radius $\left\langle {r}_g^2\right\rangle$, as defined above, bears a very important relationship to the scattering particle’s form factor *P*(*θ*), discussed earlier. Specifically, the form factor may be written (Kratochvíl 1987) as

12 $$ P\left(\theta \right)=\frac{1}{n^2}\sum \limits_{i=1}^n\sum \limits_{j=1}^n\frac{\sin \kern.2em \mu {h}_{ij}}{\mu {h}_{ij}}=\frac{1}{n^2}\sum \limits_{i=1}\sum \limits_{j=1}\left[1-\frac{{\left(\mu {h}_{ij}\right)}^2}{3!}+\frac{{\left(\mu {h}_{ij}\right)}^4}{5!}-...\right], $$

where $\mu =\frac{4\pi }{\lambda}\sin \frac{\theta }{2}.$ From Eq. (8), we can rewrite Eq. (12) to obtain

13 $$ {\displaystyle \begin{array}{l}P\left(\theta \right)=1-\frac{\mu^2}{3.2.{n}^2}\sum \limits_{i=1}^n\sum \limits_{j=1}^n\left({h}_{ij}^2\right)+\frac{\mu^4}{\mathrm{5.4.3.2}{n}^2}\sum \limits_{i=1}^n\sum \limits_{j=1}^n\left({h}_{ij}^4\right)-...\\ {}\\ {}\kern2em =1-\frac{\mu^2}{3}\left\langle {r}_g^2\right\rangle +\frac{\mu^4}{\mathrm{5.4.3.2}{n}^2}\sum \limits_{i=1}^n\sum \limits_{j=1}^n\left({h}_{ij}^4\right)-...\end{array}} $$

where ${\mu}^2=\frac{16{\pi}^2}{\lambda^2}{\sin}^2\left(\theta /2\right)=\frac{16{\pi}^2}{\lambda^2}\xi .$ Differentiating Eq. (13) with respect to *ξ*, where *ξ* = sin^2^(*θ*/2), yields the relation between $\left\langle {r}_g^2\right\rangle$ and the variation of *P*(*θ*) with respect to ξ viz.

14 $$ \left\langle {r_g}^2\right\rangle =\frac{- dP\left(\theta \right)}{d\xi}\left(\frac{3{\lambda}^2}{16{\pi}^2}\right)+\mathrm{terms}\propto {\xi}^2,{\xi}^4,... $$

If we now restrict Eq. (14) to the *initial* slope of *P*(*θ*), i.e., $\underset{\xi \to 0}{\lim}\frac{d P\left(\theta \right)}{d\xi},$ we obtain the familiar result

15 $$ \left\langle {r_g}^2\right\rangle =\left(\frac{3{\lambda}^2}{16{\pi}^2}\right)\underset{\xi \to 0}{\lim}\left(\frac{- dP\left(\theta \right)}{d\xi}\right). $$

**Fig. 7:**
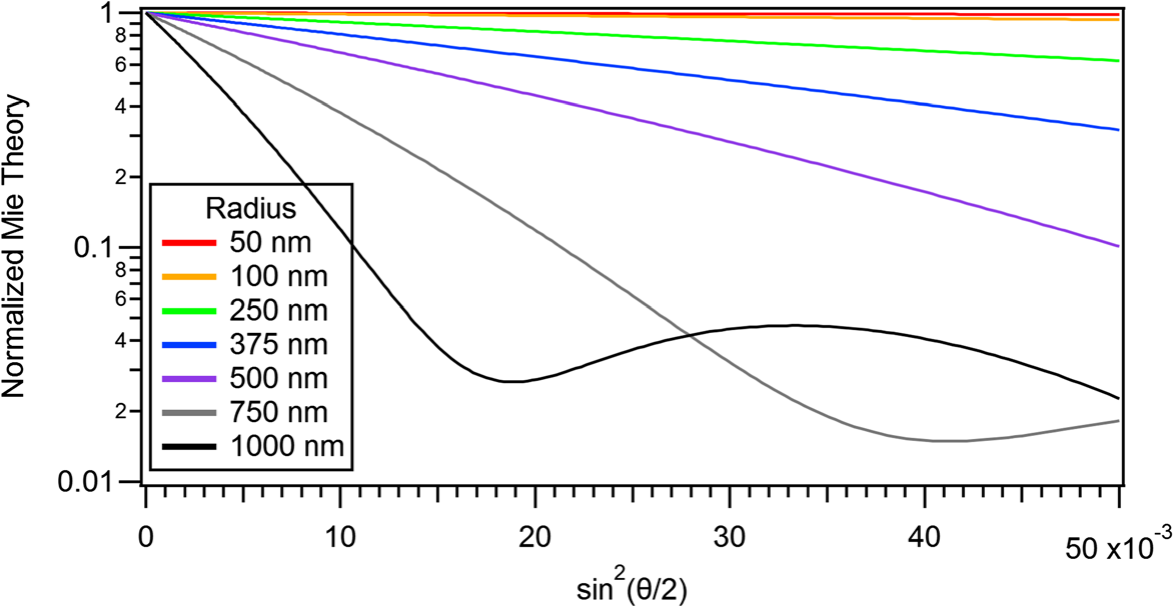
Scattering calculated at very small angles by PSL spheres in water for radii between 50 and 1000 nm at an incident wavelength of 664 nm

Kratochvil (1987), among others, has emphasized that this result applies to any particle shape and, therefore, *particle size determination based on light scattering is unique*. By measuring the *slope* of the scattering at *very small scattering angles* of an ensemble of identical particles, the mean square radius may be determined and, from it, a physical dimension of the particles may be derived. It is important to remember, however, that all of these conclusions relate explicitly to the RG approximation and its limits of applicability. Equation (15), i.e., determining $\left\langle {r}_g^2\right\rangle$ by measuring the initial slope [with respect to *ξ* = sin^2^(*θ*/2)], represents a conceptually simpler means for determining the size of homogeneous spheres in the RG approximation than using a non-linear least squares fit of the collected data to Eq. (7) and, even more certainly, for determining the length of homogeneous rods given their diameter than using a non-linear least squares fit of the collected data to Eq. (5). Equation (15) has been a long recognized consequence of determining $\left\langle {r}_g^2\right\rangle$ in the RG approximation. The major difficulty of applying this method lies universally in the fact that measurements at sufficiently low angles are rarely possible and, when they are, they may not be sufficiently precise. Figure 7 below confirms that even for simple spherical particles with negligible experimental noise; the small angle measurements needed to make use of the apparent simplicity of Eq. (15) are not generally available in commercial instrumentation.

We now emphasize one of *the major objectives of this paper*: Determination of the values of $\underset{\xi \to 0}{\lim}\frac{d P\left(\theta \right)}{d\xi}$ based on measurements traditionally made by light scattering instrumentation in the range shown in Table 1. In the early applications of light scattering to polymer chemistry, the derived values of $\left\langle {r}_g^2\right\rangle$ were relatively small since the polymer analyses were focused almost exclusively on molecules whose size was generally much less than the wavelength of the incident illumination provided by the Hg-arc lamps of that period (Zimm 1948a, 1948b). Historically, however, with particular interest in a variety of large spherical particles whose sizes were often comparable or even larger than the wavelength of the incident radiation, there were considerable efforts made (Latimer and Tully 1968; Mullaney and Dean 1969) in trying to fit collected data to the LM theory or even the RG approximation to derive size and structure information from MALS measurements made at very small scattering angles less than the first minimum. Unlike small scattering angle measurements of small particles where contaminating background debris often would affect such measurements, for large particles, their forward scattered intensities generally overwhelmed most noise contributions that might have been present.

## Analytical extension: the form factor at very small angles

Shown in Fig. 7 is a set of scattering results at very small scattering angles [$0<{\sin}^2\left(\frac{\theta }{2}\right)<0.05$, i.e., 0 < *θ* < 26°] from the exact LM theory for a set of polystyrene latex spheres in water of radii from 50 to1000 nm at an incident wavelength of 664 nm. Note that an accurate determination of the initial slope for all such sizes usually would require measurements at very low angles, though for small particles background debris may play a major disruptive roll. On the other hand, large particle size determinations require very precise measurements at small angles where significant slope deviations are prone to slight errors in the angular measurements themselves.

For measurement of light scattered from an ensemble of identical scattering particles, in order to derive useful particle size information beyond that derived traditionally from the RG approximation, we will require three specific items: (i) the structure of the particles present in the sample being measured (e.g., tubes and disks), (ii) a function that represents the scattering by an ensemble of such identical particles averaged over all orientations with respect to the direction of the incident illumination, whose variation as sin^2^(*θ*/2) → 0 will correspond to a form factor *similar* to the RG form factor *P*(*θ*), and (iii) the slope at *ξ* = sin^2^(*θ*/2) = 0 of the function that represents the ensemble scattering. Recall from the analyses of Kerker et al. (1963), that the RG approximation more closely agrees with the correct scattering formalism (in that case, the LM theory) as the scattering angles measured become smaller. Few exact theories for other structures, such as the LM theory for homogeneous spheres and similar spherically symmetric structures, have been developed explicitly, so we must seek a best fit of the scattering data to an analytical function per item (*ii*) from which $\left\langle {r}_g^2\right\rangle$ would be derived from its initial slope per item (*iii*).

In Fig. 2, for example, the scattered intensities, *I*(*θ*_*i*_) (*i* = 1, 15), are shown as collected at *n* = 15 distinct values of sin^2^(*θ*_*i*_/2) from the ensemble of identical single-wall carbon nanotubes. The best fit to the thin rod structure per Eq. (6) is shown. Although these single-wall carbon nanotubes are only of diameter 1.2 nm, the thin rod model, so commonly used, *does not fit the data well and does not, therefore, appear be an appropriate model of their scattering*. We assume, of course, that the A4F process (Podzimek 2011) yields identical lengths in each fractionated slice. The errors associated with those 15 data are very small, so we conclude that these measurements themselves are representative of the analytical scattering function that the actual particles would produce were such available.

We now attempt to generate a function equivalent to the RG function *P*(*θ*) analytically. Note from Eq. (4) that the recorded scattered intensity *I*(*θ*) is proportional to the form factor *P*(*θ*) whose behavior near sin^2^(*θ*/2) = 0 we hope to derive per item (ii) above. We begin by fitting the collected data to a polynomial of order *m* in *ξ* = sin^2^(*θ*/2) viz.

16 $$ {f}_m\left(\xi \right)=\sum \limits_{i=0}^m{\left(-1\right)}^i{c}_i{\xi}^i,m\le n-1. $$

From Eq. (16), we define the normalized function

17 $$ {\varPi}_m={f}_m\left(\xi \right)/{c}_0. $$

*Π*_*m*_(0^∘^) = 1 as we confirm per Eq. (18), below. Note that *Π*_*m*_(*ξ*) is a functional representation (through a power series) of the *actual measurements*. Finally, we assume that in the limit as *ξ* = sin^2^(*θ*/2) → 0,

18 $$ P\left(\theta \right)\to \underset{\xi \to 0}{\lim }{\varPi}_m\left(\theta \right)={f}_m\left(\xi \right)/{c}_0=1-{c}_1\xi /{c}_0+{c}_2{\xi}^2/{c}_0-... $$

However, from Eq. (15), $\left\langle {r_g}^2\right\rangle =\left(\frac{3{\lambda}^2}{16{\pi}^2}\right)\underset{\xi \to 0}{\lim}\frac{-d{\varPi}_m\left(\theta \right)}{d\xi}.$ Therefore $\left\langle {r_g}^2\right\rangle =\left(\frac{3{\lambda}^2}{16{\pi}^2}\right)\underset{\xi \to 0}{\lim}\frac{-d{\varPi}_m\left(\theta \right)}{d\xi},$ or simply

19 $$ \left\langle {r_g}^2\right\rangle =\underset{\xi \to 0}{\lim}\frac{-d{\varPi}_m\left(\xi \right)}{d\xi}\left(\frac{3{\lambda}^2}{16{\pi}^2}\right)=\frac{c_1}{c_0}\left(\frac{3{\lambda}^2}{16{\pi}^2}\right). $$

The association of the “traditional” RG form factor *P*(*θ*) with the limiting form of the calculated function *Π*_*m*_(*ξ*) in Eq. (19) is a consequence of Eq. (3). It remains, therefore, to determine the polynomial degree *m* of the Eq. (16) that will fit best the measured data and to select a model by which the derived $\left\langle {r}_g^2\right\rangle$ may be used therefrom to determine explicit particle dimensions.

Traditionally, multiangle light scattering (MALS) measurements of spherical particles are interpreted by performing a non-linear least squares fit of the collected data to an assumed structure such as a homogeneous sphere or perhaps even a radially symmetric form (Wyatt 1962) understood or confirmed by other physical measurements such as electron microscopy. Particles of more complex structure are by far the most common (ellipsoids, thick rods, tubes, and aggregates of spheres) and there are virtually no explicit analytical forms corresponding to averaging them over all relative orientations. From the actual data collected (such as the data points shown in Fig. 2), we create function *Π*_*m*_(*θ*) that best represents the collected data and, from which, we hope to derive its mean square radius $\left\langle {r}_g^2\right\rangle$.

## Examples of the analytical representation by *Π*_*m*_(*θ*)

The question now arises as to what order *m* of the function *Π*_*m*_(*ξ*) will yield the “correct” or, at least, the best value of the mean square radius of the sample measured? If *Π*_*m*_(*ξ*) is fit to the data collected at *p* of the different angles of Table 1, is there a “best fit”, i.e., a number *m* where 0 < *m* < *p*, that will yield the most accurate value for $\left\langle {r}_g^2\right\rangle$? Historically, this question was never addressed for the molecules considered by Zimm (1948a, 1948b) nor whenever measurements were made from light scattered by small particles, such as proteins. The order was assumed always to be 1. Most light scattering results presented usually produced a value of *r*_*g*_ without further comment on its relation to the structure of the specific particles/molecules measured. As we shall see presently, there is a basis for selecting a value of *m* that will yield a best fit of the data collected from measurements at *p* angles.

**Fig. 8:**
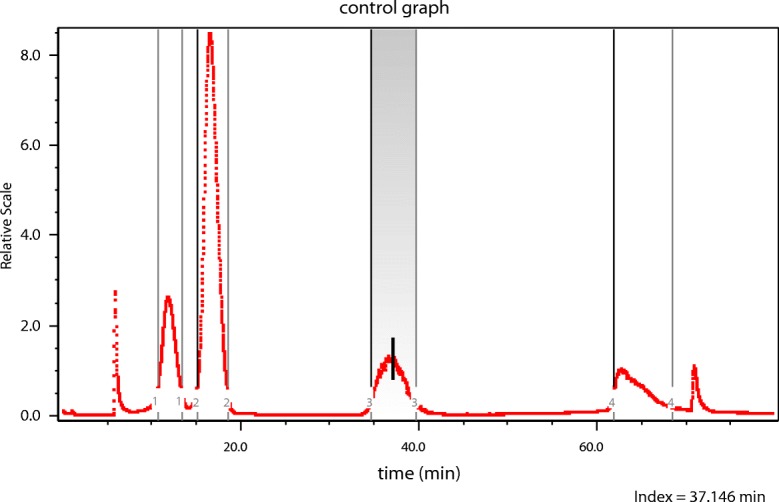
The elution profile (90° scattering vs. time) of 3 PSL samples following fractionation

**Fig. 9:**
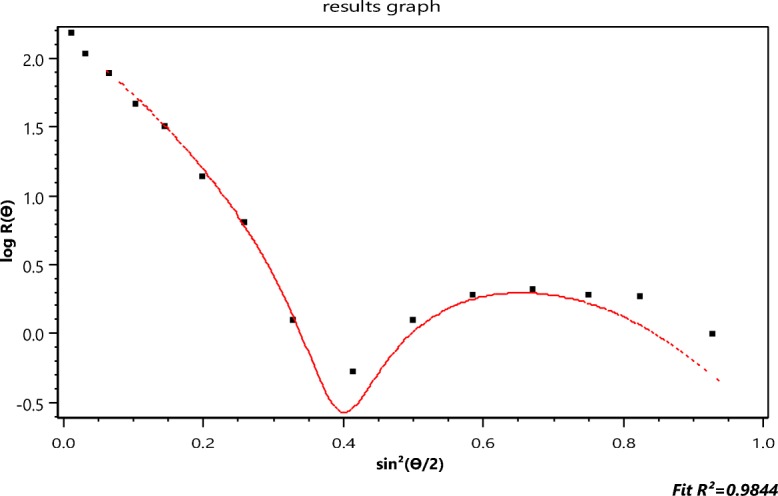
The collected scattering data for slice 2207 with their best fit to the LM theory.log[*R*(*θ*)]*vs*. sin^2^(*θ*/2)

**Fig. 10:**
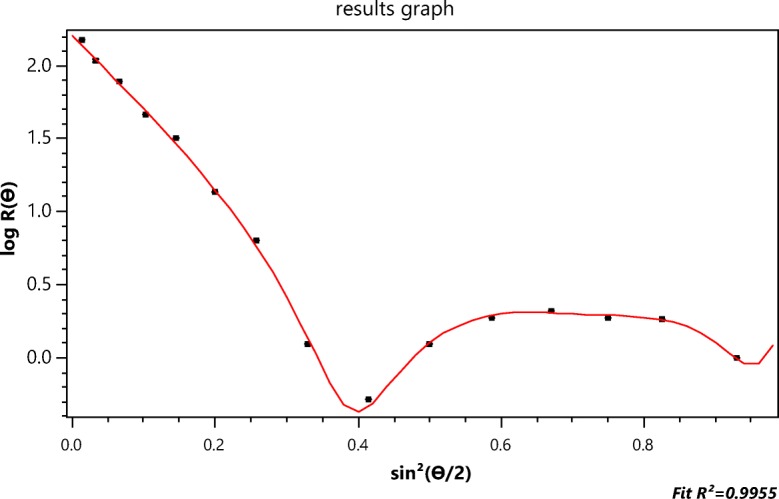
Fit of the function *Π*_8_ to the data of Fig. 8. log[*R*(*θ*)]*vs*. sin^2^(*θ*/2)

To illustrate this result, consider the fractionation and subsequent measurements of a mixture of three sizes of PSL spheres (diameters 50, 100, and 500 nm) as shown below in Fig. 8. The shaded area/peak of Fig. 8 corresponds to the separated 500-nm fraction. Slice (2207) is indicated by the vertical mark in the shaded peak 3 region near the 40-min elution. As indicated by the very fine vertical lines, that peak includes 296 slices from #2055 to #2351. The data collected at slice 2207 are shown in Fig. 9 with their best fit to the LM theory indicated by the continuous line, corresponding to a radius value of 253.7 ± 3.3 nm. Figure 10 presents a plot of the fit of *Π*_8_ to the same data yielding a value of *r*_*g*_ of 231.7 ± 22.4. From Eq. (11), therefore, $a=\sqrt{5/3}{r}_g=293\pm 28.$ Note the anomalous shape of the right peak of Fig. 10. Finding the “best fit” value of *m* is now a straightforward consequence of the results tabulated in Table 2, below. Consider the radii associated orders 3 ≤ *m* ≤ 8 as shown. Associated with each order is a derived *r*_*g*_ value and its derived uncertainty. The ratios of the experimental uncertainties, *Δr*_*g*_, to their derived values *r*_*g*_ are shown also. The best fit corresponds to the smallest relative order, i.e., the smallest ratio, 0.003, for these orders, i.e., *m* = 5 yielding a radius of 255 nm. Note that this is also the closest value to the best fit of the data to the applicable LM theory, i.e., 253.7 ± 3.3 nm of Fig. 9. For polystyrene spheres in water, ∣*m* − 1 ∣  = 0.2 and one would expect good agreement between LM and RG theories.

**Fig. 11:**
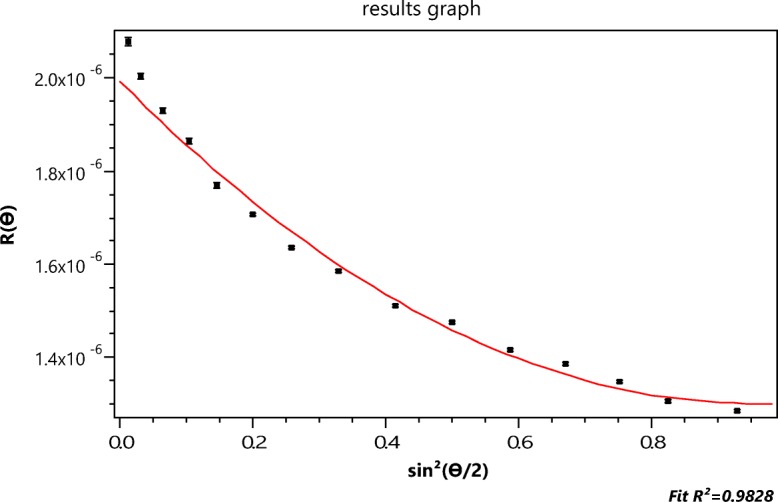
The data of Fig. 2 compared to the fit of function *Π*_2_ yielding an *r*_*g*_ of 58.8 ± 2

**Table 2:** The derived values for the polystyrene particles of *r*_*g*_, Δ *r*_*g*_, the ratio Δ *r*_*g*_/*r*_*g*_ and the derived radii $a=\sqrt{5/3}{r}_g$

*m*	*r*_*g*_	*Δr*_*g*_	Ratio	Radius nm
3	157.1	20.7	0.132	199 ± 27
4	183.3	9.7	0.053	232 ± 13
5	201.5	5.1	0.025	255 ± 6.6
6	208	9.4	0.045	263 ± 12
7	220.2	14	0.064	279 ± 18
8	231.7	22.4	0.097	293 ± 29

Although the data at the particular slice 2207 in the region of peak 3 appear consistent with the LM result, will the other slices in this peak 3 region be similar or will the optimal order vary with the small size differences within the peak? Table 3 compares the derived optimal *r*_*g*_ values for the listed six slices spanning most of the peak 3 region of Fig. 8 and confirms that the formalism associated with the determination of the optimal fit order is consistent throughout the peak.

**Table 3:** The ratios of *Δr*_*g*_/*r*_*g*_ in the vicinity of order 5 in peak 3

Peak	3			
Slice#	Order	Ratio	*r*_*g*_	time(min)
2090	4	0.055		35.176
	5	0.028	201.6	
	6	0.052		
2120	4	0.053		35.68
	5	0.024	201.5	
	6	0.042		
2200	4	0.053		37.028
	5	0.026		
	6	0.048		
2260	4	0.055		38.038
	5	0.026	201.5	
	6	0.047		
2280	4	0.053		38.375
	5	0.025	201.6	
	6	0.046		
2300	4	0.052		38.712
	5	0.023	201.4	
	6	0.042		

For the next example, we return to Fig. 2 (the collected SWCNT data for slice 757 fit to the rod model of the RG approximation) and the determination of the function *Π*_*m*_(*θ*) that will fit these data best. The rod model of the RG scattering approximation is clearly inappropriate and would not, therefore, be expected to produce realistic estimates of the SWCNT lengths. Electron micrographs have shown that these single-wall carbon nanotubes are extremely narrow tubes (radius *a* of the order of 0.6 nm and thickness *t* that of the carbon atom 0.154 nm) yielding an effective value (Wyatt 2014) per unit length of the term ∣*m* − 1∣ of Eq. (1) to be about 0.73. Accordingly, we would expect the use of the functions *Π*_*m*_ will provide a better estimate of their length. The function *Π*_*m*_ will be used to provide the corresponding slope at the origin needed to produce a value of $\left\langle {r}_g^2\right\rangle$ per Eq. (19). The relation between $\left\langle {r}_g^2\right\rangle$ and the length *L* of a tube of radius *a* and thickness *t* is just (Wyatt 2014)

20 $$ \left\langle {r}_g^2\right\rangle =\frac{L^2}{12}+{a}^2+\frac{t^2}{2}- at $$

With lengths *L* in excess of 100 nm and the values of *a* and *t* for the nanotubes described, Eq. (20) becomes simply $\left\langle {r}_g^2\right\rangle =\frac{L^2}{12}.$ Thus $L=\sqrt{12}{\left\langle {r}_g^2\right\rangle}^{1/2}=\sqrt{12}{r}_g,$ where the *root mean square* (rms) radius ${r}_g={\left\langle {r}_g^2\right\rangle}^{1/2}.$

**Fig. 12:**
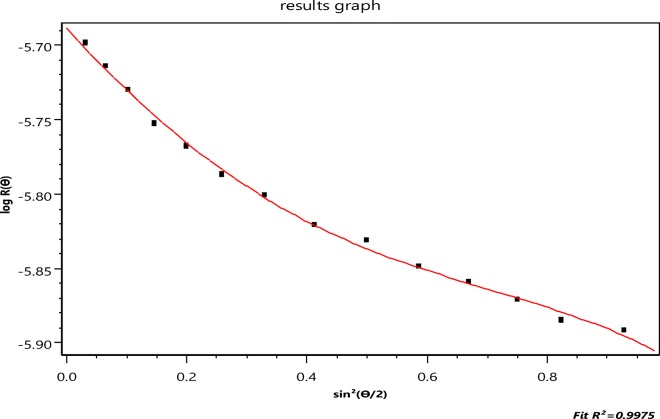
The data of Fig. 2 compared to the fit of function *Π*_3_ yielding an *r*_*g*_ of 70.7 ± 2.4

Figure 11 below corresponds to fit of the data by *Π*_2_. The corresponding *r*_*g*_ value for that slice is 58.8 ± 2, as shown. The associated value of *L* is therefore 204 ± 7 nm. This fit does not even appear more accurate than the thin rod model value of 189 nm from Fig. 2, so higher orders should be examined. Table 4 similar to Table 2, presents all values for *m* ≤ 7 from which we hope to find the most appropriate representation. Applying the same criterion used previously to determine the best value of *m* representative of the scattering by the 500 nm spheres per Table 2, we note immediately that the best functional representation of the SWCNT data from slice 757 of the elution corresponds to *Π*_3_, i.e., *m* = 3 with an associated length of 245 ± 8 nm well within the range expected after filtration of SWCNTs to lengths less than 450 nm. This comparison is shown in Fig. 12.

**Table 4:** The derived values for the SWCNT sample slice 757 of *r*_*g*_, Δ*r*_*g*_, the ratio Δ*r*_*g*_/*r*_*g*_, and the derived length $L=\sqrt{12}{r}_g$

m	*r*_*g*_	*Δr*_*g*_	Ratio	Length nm
1	42.2	1.7	0.040	146 ± 6
2	58.8	2.0	0.034	204 ± 7
3	70.7	2.4	0.014	245 ± 8
4	81.6	2.4	0.029	283 ± 8
5	81.0	4.7	0.060	281 ± 16
6	86.3	7.6	0.088	299 ± 26
7	78.9	13.8	0.175	273 ± 48

We now return to the aggregated spheres’ data of Fig. 5 in order to derive the mass of typical particles in the *second* peak that eluted around 37 min. Unfortunately, there will be larger particles present in that peak due to the huge aggregates to the right. So if we settle on slice 2193 shown eluting around 36.9 min, we obtain *r*_*g*_ = 60 + 8. (The mean square radii for six different aggregates of identical spheres are listed in Fig. 1 by Wyatt (2014) together with those of 10 other structures. For the dimer, $\left\langle {r}_g^2\right\rangle =8/5{a}^2$.) Assuming a dimer, $a={r}_g\sqrt{5/8}=$0.79(60 ± 8) = 48 ± 6 nm. This confirms that the second peak of Fig. 6 corresponds to the second peak from the right of Fig. 5, i.e., a dimer. The large errors associated with this dimer peak of Fig. 5 are associated to the very noisy data present in the A4F measurement of Fig. 6.

**Fig. 13:**
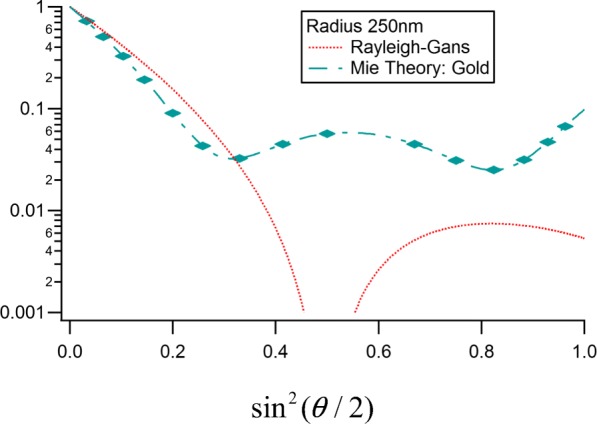
Comparison of the RG sphere model to the LM theory (Table 1 angles marked) for homogeneous gold spheres of radius 250 nm in water

Finally, we return to the 500-nm gold particles whose calculated scattering is shown in Fig. 13. Adding to these calculated values, the estimated experimental errors of the water solvent per, for example, those measured during the collection of the data shown in Fig. 8, yield Table 5 below. From Eq. (17), we have ${r}_g={\left\langle {r_g}^2\right\rangle}^{1/2}=\sqrt{3{c}_1/{c}_0}\lambda /\left(4\pi \right)=68.19\sqrt{c_1/{c}_0}.$ The smallest ratio corresponding to *Π*_5_ yields an *r*_*g*_=206 and *a* = $\sqrt{5/3}{r}_g=$266 nm; a value differing from the gold particle radius of 250 nm by less than 7%. These particles are virtually impenetrable by the incident radiation.

**Table 5:** The derived values for the gold particles of *r*_*g*_, Δ*r*_*g*_, the ratios Δ*r*_*g*_/*r*_*g*_, and the derived radii $a=\sqrt{5/3}{r}_g$

*m*	*r*_*g*_	*Δr*_*g*_	Ratio	Radius
4	193	9	0.047	249 ± 12
5	206	6.4	0.031	266 ± 8.3
6	212	13	0.061	273 ± 17
7	221	20	0.090	285 ± 26

## Summary and conclusions

Nanoparticles of greatest current interest and importance, both in regard to their physical properties and practical application, are in the size range of a few hundred nanometers, i.e., from about 50 nm to 2000 nm: the range of focus of this paper. Once a monodisperse sample is obtained of such particles in suspension (generally aqueous), and their structure is known; the determination of their size/dimension-based measurement of the scattering of monochromatic light by the ensemble becomes the basic objective of the measurement. Traditionally, the measurements obtained over a broad range of scattering angles are fit in a non-linear least squares sense to a theoretical model of how such particles will scatter incident light of defined wavelength and polarization. For the case of homogeneous spheres, the Lorenz-Mie theory is so fit to the collected data to yield their size. There are very few other structures for which such an exact theory is available except in the case when 2*ka* ∣ *m* − 1 ∣ ≪ 1 discussed in the “The Lorenz-Mie theory and the Rayleigh-Gans approximation” section as the Rayleigh-Gans approximation. Even in the limits associated with that approximation, the functions that must be fit to the measurements are often very complex [Cf. Eq. (5)]. Rather than relying upon the non-linear least squares fitting of the approximate RG formulae to the collected data, a simpler approach has been developed by which means the scattering particles’ mean square radius $\left\langle {r}_g^2\right\rangle$ is calculated and then used to derive the sought shape parameters (e.g., radius, length, and thickness) following, for example, the sets presented by Wyatt (2014). Deriving the appropriate $\left\langle {r}_g^2\right\rangle$ values based upon a set of form functions *Π*_*m*_(*θ*), similar to the familiar form factors *P*_*m*_(*θ*), has been described. Key to selecting the best order of *Π*_*m*_(*θ*) is the determination of the order producing the smallest relative error of the ratio $\varDelta {r}_g/{\left\langle {r}_g^2\right\rangle}^{1/2}.$ Following the determination of the optimal ${\left\langle {r}_g^2\right\rangle}^{1/2},$ and with the a priori knowledge of the particles’ shape and its dependence on ${\left\langle {r}_g^2\right\rangle}^{1/2},$ the particles’ structural properties are calculated. Such a procedure, even for particles well described by the RG approximation at all angles, represents the simplest means to determine such structural parameters in contrast, for example, to using the collected data to extract such information by performing a non-linear least squares fit to expressions such as Eq. (7) for simple spheres, or even Eq. (5) for cylinders.

The determination of the best fit order *m* and the subsequent application of Eq. (19) to determine the particles’ size parameters (using the known relations between ${\left\langle {r}_g^2\right\rangle}^{1/2}$ and these parameters as listed, for example, for several types of particles by Wyatt [2014]) represent the most important consequences of this paper. The paper confirms also that for a broad range of particles sizes well outside of the strictures of Eqs. (1) and (2), very good approximations of their sizes may be obtained with relative ease and simplicity. Great efforts have been expended historically (Sharma and Somerford 2006) to obtain means by which particle size features may be extracted from a variety of interpretations of their scattering properties. Again, the methods disclosed in the present paper appear to be the simplest and probably the most accurate means to achieve these goals. The derivations of Eqs. (13) and (15) appear only rarely in physics texts as their origins go far back to the work of Zimm (1948a, 1948b) and similar polymer chemistry investigations (Huglin 1972; Kratochvíl 1987). Nevertheless, it should be apparent from the present paper that the methods presented based on Eq. (19) are by far the simplest (and probably the most accurate) means to measure the size of monodisperse distributions of particles in suspension.

The need to produce a collection of monodisperse particles for subsequent application of the methods developed in this paper invariably has required means to fractionate samples of initially broad distributions. In this regard, asymmetric flow field flow fractionation (A4F) has become the preferred method. One of the most important classes of nanoparticles addressed in this paper has been single-wall carbon nanotubes (SWCNT). The process by which FFF actually produces monodisperse subsets of SWCNTs has been an area of particular interest and application for well over a decade, yet remains poorly understood. The separation of oxidatively shortened SWCNT (as well as multiwall samples) using the more difficult cross flow FFF was reported in 2002 in a study by Chen and Selegue (2002). Their methods of measuring the fractions using both SEM and TEM were not particularly successful. Later studies by Nguyen et al. (2015) focused on high aspect ratio gold rods. It should be noted, however, that the aspect ratios of such rods are much larger than the far more important SWCNT (as well as the multi wall CNTs). No associated MALS measurements have been reported.

Despite some uncertainties of the actual mechanisms by which rod-like particles are separated in A4F devices (Park and Mittal 2015; Nguyen et al. 2013), under a variety of conditions, separations by length actually do occur [Cf. Fig. 2]. A major question then arises: What is the *actual length of the particles* within the fraction (“slice”) whose differential light scattering variation (aka MALS) is measured? At present, in order to produce an associated size, the particles/rods producing the scattering data must be collected and measured. Generally, there are two types of measurements possible: (1) by microscopic examination, one-particle-at-a-time [electron, transmission, or atomic force microscopy] or (2) by single particle inductively coupled mass spectrometry (sp ICP-MS). No wonder the thin rod model of Eq. (5) is so often used! Its results, of course, are always erroneous since they never fit SWCNT data.

Insofar as SWCNTs are concerned, their measurement, and the derived distributions of their sizes anticipated by the present paper, will require particle microscopy measurements of the quality so well described and documented by Nguyen et al. (2015) for gold rods. Thus the SWCNT particles separated at each slice, as indicated by the slice profile example of Fig. 3, should be similarly collected and measured. Such results would then be compared directly with the values at each slice calculated by the enhanced RG approximation described in the present paper. This proposed measurement program for a single sample aliquot is an essential requirement in order to confirm the interpretive model proposed. It is important to note that every A4F measurement performed using a sample whose preparation such as cited above by Nguyen, J. Liu, and V. Hackley generally requires a variety of preparative procedures including filtration and sonication. In order to confirm the applicability of the interpretive procedures described in the current paper, transmission electron microscopy measurements of the particles/rods present in each collected slice must be performed. Only by such repeated measurements will the postulated methodology of the current paper become accepted and useful. The Hackley group has produced a variety of interesting papers (Nguyen et al. 2013; Gigault et al. 2014; Cho and Hackley 2010) relating to the A4F separation process itself, though the companion use of MALS as a means for sizing gold particles or SWCNTs is not addressed.

The surprisingly good results of the derived size of large gold particles earlier in this article (within 7%) for a structure certainly antithetical to the extensive application of the RG approximation suggest that for particles of such great refractive index, the incident waves do not penetrate significantly into the particles and we are, in effect, just seeing diffraction with no dependence on internal particle structure.

Once the A4F fractionation and the accompanying sizing methodology for SWCNT and gold particles as developed in the current paper has been confirmed, an entirely new set of applications and opportunities for these particles will be possible. The ability to produce and retain for future study/use aliquots of *well-defined sizes* without the need for cumbersome microscopy and/or mass spectroscopy will have extensive application, especially in areas for medical application (Sharma and Somerford 2006; Zhang et al. 2010; He et al. 2013) of these particles.

Finally, a most important result of this study is *restated*: Using the collected multiangle scattered light signals from a suspension of monodisperse particles, the size of such particles may be determined from the initial slope [with respect to *ξ* = sin^2^(*θ*/2)] of the derived form factor *Π*_*m*_(*θ*). There should no longer be a need to perform non-linear least squares fitting of collected multiangle scattering data to any RG model [e.g., Eq. (5)] in order to obtain the associated size features of the monodisperse scattering particle.
